# Abnormal expression of *SLIT3* induces intravillous vascularization dysplasia in ectopic pregnancy

**DOI:** 10.7717/peerj.14850

**Published:** 2023-02-10

**Authors:** Qian Zhu, Xiaoya Zhao, Duo Zhang, Wei Xia, Jian Zhang

**Affiliations:** 1 International Peace Maternity and Child Health Hospital, School of Medicine, Shanghai Jiao Tong University, Shanghai, China; 2 Shanghai Municipal Key Clinical Specialty, Shanghai, China

**Keywords:** Ectopic pregnancy, Villus capillary, miRNA–mRNA network, Bioinformatics analysis

## Abstract

**Objective:**

To investigate whether the morphology, capillary number, and transcriptome expression profiles of ectopic pregnancy (EP) villi differ from those of normal pregnancy (NP) villi.

**Methods:**

Hematoxylin-eosin (HE) and immunohistochemistry (IHC) staining for CD31 were conducted to compare differences in morphology and capillary number between EP and NP villi. Differentially expressed (DE) miRNAs and mRNAs were determined from transcriptome sequencing of both types of villi and used to construct a miRNA–mRNA network, from which hub genes were identified. Candidate DE-miRNAs and DE-mRNAs were validated by quantitative reverse transcription (qRT)-PCR. Correlations were identified between the number of capillaries and serum beta human chorionic gonadotropin (*β*-HCG) levels and between the expression levels of hub genes associated with angiogenesis and *β*-HCG levels.

**Results:**

The mean and total cross-sectional areas of placental villi were significantly increased in EP compared with NP villi. Capillary density was greatly reduced in EP villi and was positively correlated with *β*-HCG levels. A total of 49 DE-miRNAs and 625 DE-mRNAs were identified from the sequencing data. An integrated analysis established a miRNA–mRNA network containing 32 DE-miRNAs and 103 DE-mRNAs. Based on the validation of hub mRNAs and miRNAs in the network, a regulatory pathway involving miR-491-5p–*SLIT3* was discovered, which may have a role in the development of villous capillaries.

**Conclusion:**

Villus morphology, capillary number, and miRNA/mRNA expression profiles in villous tissues were aberrant in EP placentas. Specifically, *SLIT3*, which is regulated by miR-491-5p, may contribute to the regulation of villous angiogenesis and was established as a putative predictor of chorionic villus development, providing a basis for future research.

## Introduction

Ectopic pregnancy (EP), in which a fertilized ovum is implanted outside of the uterine cavity, accounts for 1–2% of all human pregnancies. Ninety-eight percent of EPs occur in the fallopian tube, a phenomenon known as tubal EP (Shaw et al., 2010). Hemorrhaging as a result of EP is a major cause of maternal mortality in the first trimester, accounting for ∼10% of all pregnancy-related deaths (Farquhar, 2005).

The placental villi at the maternal-fetal interface are the bridge between mother and fetus and are therefore extremely important in embryo implantation and development (Cakmak & Taylor, 2011; Kokawa, Shikone & Nakano, 1998). Structural abnormalities or dysfunction of the villi have been implicated in pathological pregnancies, including preeclampsia, fetal growth restriction (FGR), and recurrent miscarriage (Tian et al., 2016). In tubal EP, the epithelial cells of the fallopian tube cannot undergo adequate decidualization to produce an appropriate microenvironment to support embryonic growth (Liu et al., 2020b), and most EP villi will be dysplastic or end with abortion (Cunningham et al., 2008). In a previous study, we showed that EP villi exhibit excessive oxidative stress, decreased mitochondrial DNA copy number, and mitochondrial dysfunction compared to normal pregnancy (NP) villi (Huang et al., 2020). Because mitochondria are the intracellular “energy factories”, mitochondrial dysfunction leads to metabolic dysfunction, triggers embryonic apoptosis, and prevents embryonic development (Guerin, Mouatassim & Menezo, 2001). Taken together, this evidence suggests that EP villi are dysplastic compared to NP villi, but the exact mechanism involved remains unclear.

Angiogenesis and vasculogenesis in placental villi are essential for the growth of trophoblast cells and the maintenance of NP because there is diffusion between the outer surface of the villi trophoblast and the inner surface of the villi vascular endothelium (Burton et al., 2009). Various factors, such as vascular endothelial growth factor (VEGF), acidic and basic fibroblast growth factor (aFGF and bFGF, respectively), and epidermal growth factor (EGF), reportedly affect angiogenesis and vasculogenesis through autocrine or paracrine mechanisms; these factors directly or indirectly stimulate endothelial precursor cell differentiation and proliferation, influencing angiogenesis and vasculogenesis and affecting the pregnancy outcome (Demir, Seval & Huppertz, 2007). However, it is unclear whether abnormalities in angiogenesis are responsible for dysplasia of EP villi.

To address these questions, we here compared villus morphology and capillary number between villi from EP and NP patients. Furthermore, we compared the transcriptome expression profiles of the two types of villi and identified key factors influencing EP villi angiogenesis through biological information analysis and experimental validation. This study provides new insights into the molecular mechanism underlying villous dysplasia in the tubal EP environment.

## Material and Methods

### Clinical tissue sample collection

**Figure 1:**
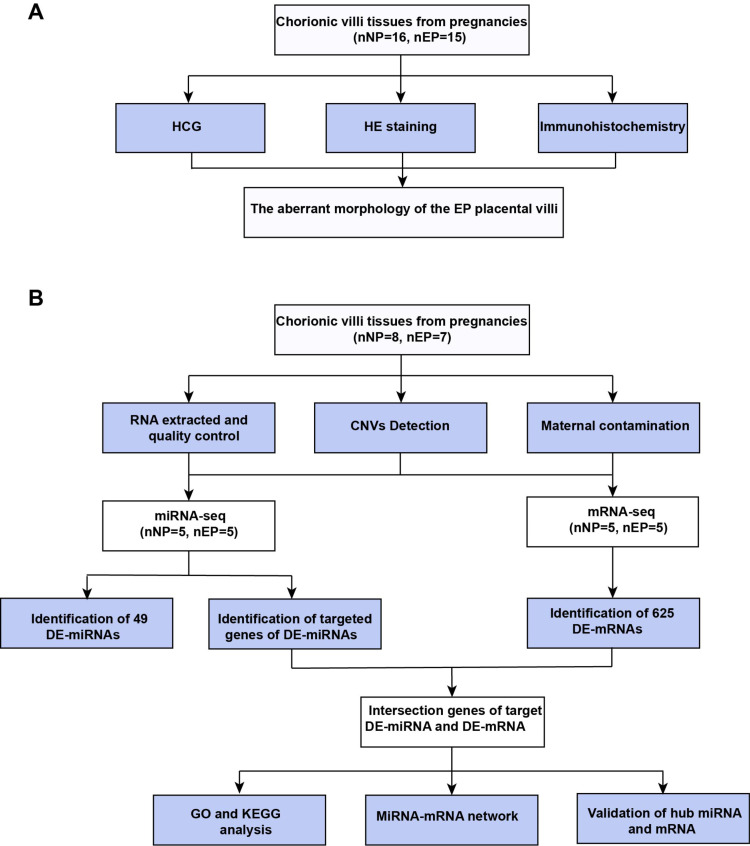
Workflow of experiments and analyses. (A) Workflow of morphology assessment between NP and EP. (B) Workflow of molecular analysis.

The experimental workflow used in this study is shown in Fig. 1. This investigation was approved by the Institutional Ethics Committee of the International Peace Maternity and Child Health Hospital (IPMCH) in Shanghai, China (GKLW201909). Written informed consent was obtained from all enrolled patients. The study was conducted between May 2020 and March 2021. Villous tissues samples were collected from women undergoing laparoscopic salpingectomy for tubal EP and women electively terminating clinical NP for nonmedical reasons. All participants had singleton pregnancies and regular menstrual cycles, and were between six and eight gestational weeks as confirmed from the time of the last menstrual period. Tissue samples were collected immediately after surgical removal of the villi, stored in liquid nitrogen or 10% formalin, and transported from the operating theatre to the laboratory within 15 min. Voluntarily terminated NPs were confirmed by ultrasonography combined with serum levels of beta human chorionic gonadotropin (*β*-HCG). The diagnosis of tubal EP was first made by ultrasound combined with serum *β*-HCG, then confirmed and treated *via* laparoscopic salpingectomy. All EP samples were obtained without methotrexate treatment. Exclusion criteria included smoking, abnormalities in vital organ function or metabolic function (*e.g.*, diabetes or obesity), hypertension or other cardiovascular pathologies, acute or chronic illness, NP with a previous history of abnormal pregnancy (*e.g.*, preeclampsia or recurrent miscarriage), EP with obvious tubal inflammatory adhesions, previous fallopian tubal diseases, or a history of tubal surgery. Detailed baseline characteristics of the samples are provided in Table S1.

### Hematoxylin-eosin (HE) staining of paraffin-embedded tissue

Paraffin-embedded samples were collected by the pathology department of IPMCH, then cut into 5-µm slices using a microtome (Leica, RM2235). HE staining was conducted following routine procedures (Liu et al., 2017). In brief, following deparaffinization and rehydration, 5-µm longitudinal sections were stained with hematoxylin solution for 5 min, immersed five times in 1% acidic ethanol (1% hydrochloric acid in 70% ethanol), then rinsed with distilled water. Sections were then stained with eosin solution for 3 min, followed by dehydration in a graded alcohol series and treatment with xylene. Slides were mounted with the synthetic resin Entellan (Merck, Darmstadt, Germany).

### Immunohistochemistry (IHC)

Using a microtome, 5-µm sections were obtained from the paraffin-embedded villous tissue blocks. The primary antibodies used here were monoclonal mouse antibody against cluster of differentiation 31 (CD31) (1:200; Abcam, Cambridge, UK, #ab9498) and rabbit polyclonal antibody against slit guidance ligand 3 (SLIT3) (1:100; Abcam, #ab198726). IHC assays were conducted by Recordbio (Shanghai, China). Tissue slices were incubated with primary antibody at 4 °C overnight, then with secondary antibody at room temperature for 50 min.

### Quantitative analysis

In CD31+ IHC sections, 30 terminal villi in each section to be analyzed were selected at random and analyzed by two independent observers blinded to the identity of the tissue groups. The following parameters were measured using Fiji software (Wen et al., 2014): mean cross-sectional area of placental villi, total cross-sectional area of placental villi, and number of capillaries within placental villi. For each sample, the number of capillaries per unit area was calculated as the number of capillaries divided by the total cross-sectional area of the placental villi.

### miRNA-seq and mRNA-seq analyses

Ten samples (five NP and five EP) were selected for miRNA-seq and mRNA-seq analyses. The study design and specific processes used for sample collection, miRNA-seq and mRNA-seq analyses, identification of the intersection between target mRNAs and differentially expressed mRNAs (DE-mRNAs), and construction of the miRNA–mRNA network is described in the Supplementary Methods. The average depth of coverage for each transcript was 20 ×. The statistical power of this experimental design was calculated in the R package ‘RNASeqPower’ v1.34.0 from five biological replicates as 0.62.

### Quantitative reverse transcription PCR (qRT-PCR)

Sixteen villous samples (eight NP and eight EP) were used to verify the sequencing results. Total RNA was isolated using TRIzol Reagent (Invitrogen, Carlsbad, CA, USA) following the manufacturer’s instructions. cDNA was prepared using the Bulge-Loop miRNA qRT-PCR Starter Kit, the QuantiNova SYBR Green PCR Kit (QIAGEN, Hilden, Germany), and the QuantStudio 7 Flex Real-Time PCR System (Thermo Fisher Scientific, Waltham, MA, USA). The relative expression levels of four hub mRNAs associated with angiogenesis and four hub miRNAs that may target *SLIT3* were validated by qRT-PCR (primers shown in Tables S2 and S3). mRNA expression levels were standardized using the endogenous control *GAPDH* and miRNA expression levels were standardized based on levels of RNA U6. Expression levels of both mRNAs and miRNAs were normalized for between-sample comparisons using the 2^−ΔΔCt^ method (Arocho et al., 2006; Sadeghi, Hojati & Tabatabaeian, 2017; Wilkins-Haug, 2009).

### Cell culture and miRNA transfection

The immortalized human trophoblast cell line HTR8/SVneo (HTR-8) was kindly provided for this study by Dr. Yi Lin (IPMCH). Cells were cultured in Dulbecco’s Modified Eagle Medium/Nutrient Mixture F12 (DMEM/F12) with 10% fetal bovine serum (FBS) and 1% penicillin/streptomycin. HTR-8 cells were transfected with retrovirus containing the control vector or an overexpression plasmid (overexpressing either miR-491-5p or miR-34a-5p) and harvested at 72 h after transfection. Detailed procedures are included in the Supplementary Methods. Cells were washed three times with ice-cold phosphate-buffered saline (PBS) and RNA was extracted for qRT-PCR as described above.

### Dual-luciferase reporter assay

Wild-type (WT) and mutant firefly luciferase reporter constructs were chemically synthesized *in vitro* by cloning a portion of the 3′ untranslated region (UTR) of *SLIT3* containing the WT predicted miR-491-5p binding site or the associated mutant into the psiCHECK-2 plasmid. T cells (293) were cultured to ∼70% confluence in six-well plates and subsequently co-transfected with control vector or luciferase reporter vector (either SLIT3 3′UTR WT or the mutant SLIT3 3′UTR Mut) and the miR-491-5p mimic or the negative control (NC). After 48 h, cells were lysed and luciferase was detected on a microplate reader (Spark 10 M; Tecan). Renilla luciferase was used as the internal reference to quantify firefly luciferase expression.

### Sprouting angiogenesis assay

To examine capillary sprouting, spheroids were generated with human umbilical vein endothelium cells (HUVECs) as previously described (Zahra et al., 2019). On the first day of spheroid formation, HUVECs were suspended in EGM2/methylcellulose medium and incubated at 37 °C for 24 h with a 20-µl dropper hanging from the lid of a 10-cm dish. Spheroids were then incubated at 37 °C for 24 h in basal medium only or with factors. Statistical analysis of HUVEC budding was performed by randomly selecting 15 spheroids and measuring the number of sprouts per spheroid (Tetzlaff & Fischer, 2018). Each experiment was repeated at least three times.

### Statistical analysis

All experiments were performed in triplicate. Statistical analyses were performed using GraphPad Prism 8 (GraphPad Software Inc., La Jolla, CA, USA). Student’s *t*-test or the Mann–Whitney *U* test was used to determine statistical significance between the EP group and NP group based on the clinical data and experimental results. Correlations were assessed with Spearman’s rank correlation. Results were considered statistically significant at *p* < 0.05. Raw miRNA-seq and mRNA-seq data were filtered using custom Perl and Python scripts. Significant differential expression was assessed at a false discovery rate (FDR) < 0.05; the thresholds for calling DE-mRNAs and DE-miRNAs were —log_2_(FoldChange)— ≥ 1 and —log_2_(FoldChange)—≥ 0.6, respectively.

## Results

### Demographic characteristics and clinical data

The characteristics of the collected chorionic villi samples used in HE staining, IHC analyses, RNA-seq, and qRT-PCR validation are summarized in Table S1. The two groups showed similarities in terms of maternal age, gravidity, parity, and Body Max Index (BMI). Interestingly, women with EP were more likely to present with lower serum levels of *β*-HCG than women with NP (*p* < 0.0001).

### Differences in morphology and capillary number between EP and NP villi

**Figure 2:**
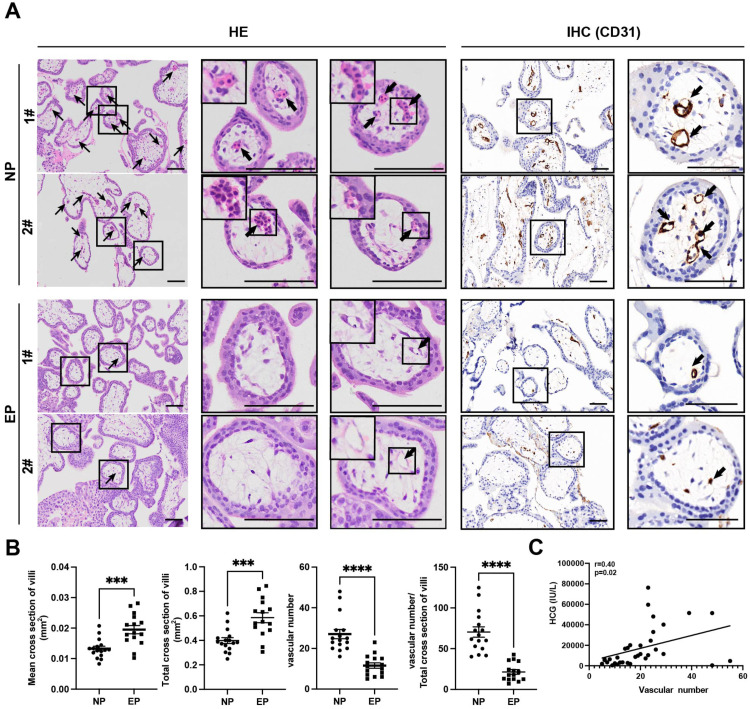
Differences in morphology and capillary number between NP and EP villi. (A). Representative images of HE (left) and IHC staining for CD31 (right) to analyze intravillous capillaries (arrows) in cross sections of NP (*n* = 16) and EP (*n* = 15) placental villi. The EP samples had fewer red cells and smaller villous cross-sectional areas in the placental villi. Scale bars, 100 µm.(B). Quantification of the mean and total cross-sectional area of placental villi, vascular numbers and the ratio of vascular numbers to total cross area of villi in NP and EP. Each dot represents a clinical sample. Both the mean and total cross-sectional area of placental villi was significantly larger in the EP sections compared to NP sections. There were fewer capillaries in the placental villi of EP sections compared to NP sections. EP villi also contained many fewer capillaries per unit area (see Methods) than NP villi. Data are represented as the mean ± standard error of the mean (SEM). (C) The number of capillaries and *β*-HCG levels were analyzed for correlation in these 31 patients (nNP = 16, nEP = 15) using the Spearman’s rank correlation test. A positive correlation between the number of capillaries and *β*-HCG levels was indicated by the results. ^∗∗∗^*p* < 0.001, ^∗∗∗∗^*p* < 0.0001. NP: normal pregnancy, EP: ectopic pregnancy. Arrows point to capillaries within the villi and squares surround placental villi.

Differences between EP and NP villous capillaries were uncovered by HE staining of tissue sections (Fig. 2A); EP samples had fewer red cells and capillaries. Furthermore, IHC staining of the vascular endothelial cell marker gene CD31 confirmed the HE staining results. Compared with NP, EP placental villi exhibited higher mean and total cross-sectional areas (Fig. 2B). Nevertheless, the relative proportions of proliferating (KI67+TEAD4+) and resting (KI67-TEAD4+) cytotrophoblasts (CTBs) were similar in the EP and NP groups, implying that stem cell population proliferation was also comparable between the two types of placental villi (Liu et al., 2018) (Fig. S2A). EP villi also contained many fewer capillaries per unit area than NP villi (Figs. 2A, 2B), and the vascular number was strongly associated with *β*-HCG levels (*r* = 0.40, *p* = 0.02) (Fig. 2C). Taken together, these observations indicated that EP villi were more poorly developed than NP villi at comparable gestational ages.

### Differences in miRNA and mRNA profiles between EP and NP villi

**Figure 3:**
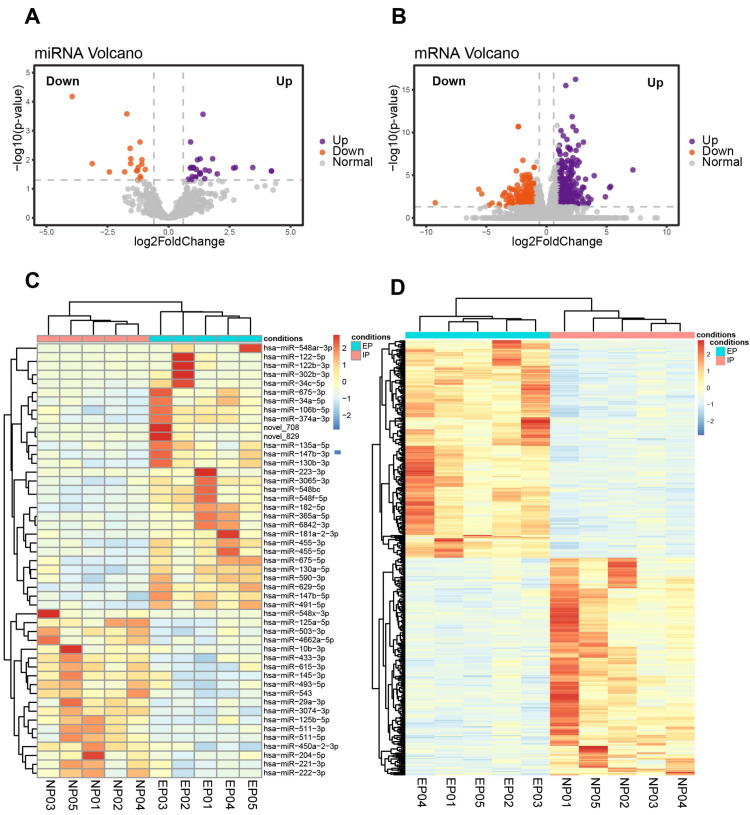
Identification of DE-miRNAs and DE-mRNAs in villus tissue from EP and NP. (A). Volcano plot showing DE-miRNAs in EP compared to NP villi, cross-referenced with -log *(p-* value) (*y*-axis) and log2 fold change (FC) (*x*-axis). Orange and purple represent down-regulation and up-regulation, respectively; gray dots indicate non-significantly changed miRNAs. All comparisons are EP *vs.* NP. (B). Volcano plot showing DE-mRNAs in EP compared to NP. —log 2FC—≥ 1 and *p*-value < 0.05 were used as the thresholds for calling significantly different expression. (C) Heatmap of DE-miRNAs among the EP and NP groups. Columns represent individual libraries, rows indicate gene symbols of DE-mRNAs or DE-miRNAs, and the color bar indicates relative expression level from high (red) to low (blue). (D) Heatmap of DE-mRNAs among the EP and NP groups.

Ten of the villous samples (five EP and five NP) were selected for miRNA- and mRNA-seq analyses. Selections were made on the basis of RNA quality, copy number variation (CNV), and lack of maternal contamination (Table S4). There were 49 significant DE-miRNAs, including 30 up-regulated and 19 down-regulated miRNAs in tubal EP villous samples (Figs. 3A, 3C, Table S5), with a predicted total of 5939 putative target genes. mRNA-seq results revealed 342 up-regulated and 283 down-regulated genes in EP compared to NP villi (Table S6). Normalized counts and log_2_(FoldChange) values were visualized with a volcano plot (Fig. 3B). Expression profiles of DE-mRNAs in the two groups were also evaluated with hierarchical clustering (Fig. 3D).

### Construction of a miRNA–mRNA network

**Figure 4:**
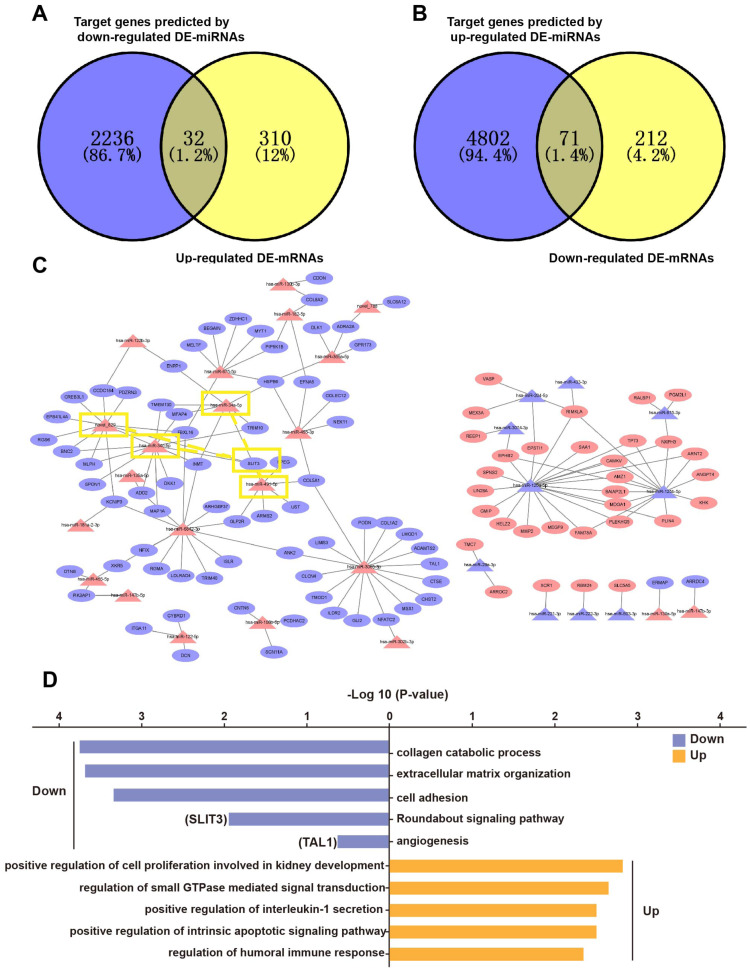
Overlapping genes between DE-mRNAs and target genes predicted by DE-miRNAs and miRNA-mRNA regulatory network. (A) Venn diagram of overlapping genes between up-regulated DE-mRNAs and predicted target genes of down-regulated DE-miRNAs. (B) Venn diagram of overlapping genes between down-regulated mRNAs and down-regulated mRNAs and predicted target genes of up-regulated DE-miRNAs. (C) miRNA-mRNA regulatory network. Red triangles represent upregulated miRNAs, purple triangles represent downregulated miRNAs, red ovals represent upregulated mRNAs, purple ovals represent downregulated mRNAs and lines represent interactions between DE-miRNAs and DE-mRNAs. The yellow rectangles indicate *SLIT3* and miRNAs that may target *SLIT3*. (D) Enriched GO terms. Purple and orange represent downregulated and upregulated expression, respectively. Among them, *SLIT3* was enriched in Roundabout signal pathway. TAL1 was also enriched in term of angiogenesis.

Based on the principle of mRNA and miRNA pairing, 32 up-regulated DE-mRNAs (Fig. 4A) and 71 down-regulated DE-mRNAs were visualized in a Venn diagram (Fig. 4B). The 103 overlapping genes were analyzed for enrichment of Gene Ontology (GO) terms (Fig. 4D) and Kyoto Encyclopedia of Genes and Genomes (KEGG) pathways (Fig. S1) to understand their biological functions in EP. The down-regulated genes were significantly enriched in the following biological processes (BPs): collagen catabolic process, extracellular matrix (ECM) organization, cell adhesion, Roundabout (*Robo*) signaling pathway, and angiogenesis. The up-regulated genes were enriched in BPs including positive regulation of cell proliferation involved in kidney development, regulation of small GTPase mediated signal transduction, and positive regulation of intrinsic apoptotic signaling pathway. In addition to the GO term of angiogenesis, the *Robo* signaling pathway has also been reported to be associated with angiogenesis and placenta vascular remodeling (Li et al., 2015; Liao et al., 2012; Zhou et al., 2011). These results illustrated that pro-angiogenesis genes (*e.g.*, *SLIT3* and *TAL1*) were down-regulated in EP villi (Hu et al., 2016; Lazrak et al., 2004; Lelievre et al., 2001; Li et al., 2015; Liao et al., 2012; Zhou et al., 2011), consistent with the observed low capillary number. In addition, in agreement with dysplasia of EP villi compared to NP villi, we also found that pro-apoptotic genes were up-regulated in EP samples. The 103 overlapping genes were regulated by a total of 32 DE-miRNAs. A regulatory network of the 103 mRNAs and 32 miRNAs was constructed (Fig. 4C).

### Validation of hub mRNAs associated with angiogenesis

**Figure 5:**
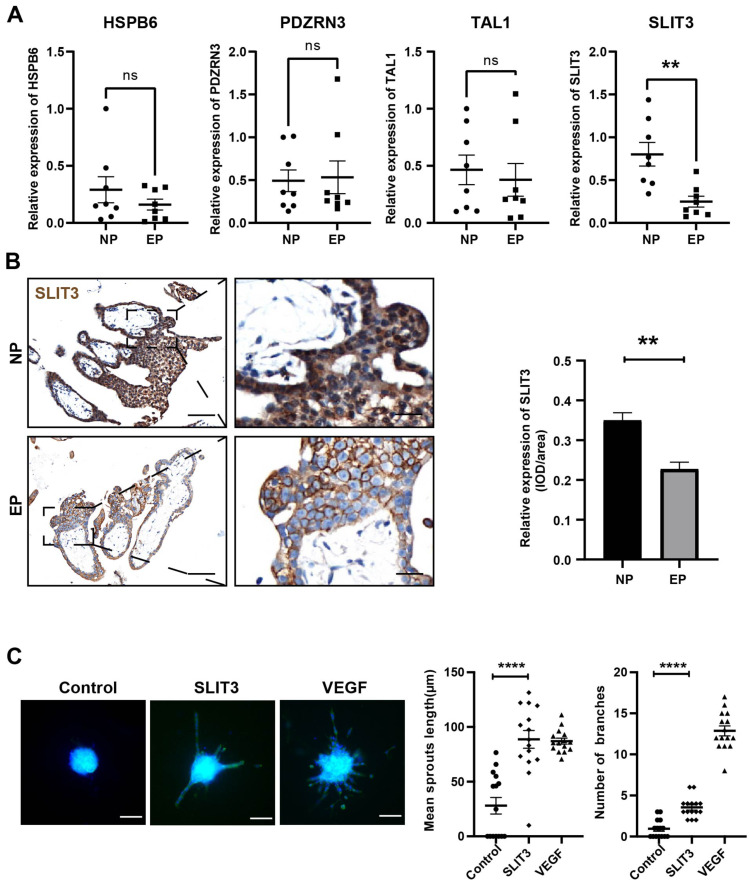
Validation of the four hub mRNAs associated with angiogenesis in the villi of EP and NE groups and the effect of *SLIT3* on angiogenesis. (A) Validation of the four hub mRNAs associated with angiogenesis in the villi of EP (*n* = 8) and NP (*n* = 8) groups *via* qRT-PCR. (B) Single staining of maternal villi using anti-*SLIT3* antibody. Left scale bar = 100 µm; right scale bar = 25 µm. IHC score values of *SLIT3* were significantly lower in the EP group (*n* = 5) compared to NP (*n* = 5). ^∗∗^*p* < 0.01, ns, no significance. (C) The angiogenic potential of *SLIT3* is tested by sprouting assays. VEGF (250 ng/ml) was used as a positive control. A concentration of 6 µg/ml of *SLIT3* was chosen with reference to literature reports (Greaves et al., 2014). Each data point is a quantification of 15 HUVEC spheres (field of view) for each culture medium. All experimental groups were repeated at least three times and a representative result was shown. Scale bar =100 µm. ^∗^ *p* < .05, ^∗∗^ *p* < .01, ^∗∗∗^ *p* < .001, ^∗∗∗∗^*p* < .0001. NC, Negative control. Data are represented as mean ± SEM.

Based on the GO term analysis and a review of the literature, we found that four of the 103 differentially expressed genes were associated with angiogenesis, namely *HSPB6* (Zhang et al., 2012), *TAL1* (Hu et al., 2016), *PDZRN3* (Sewduth et al., 2017), and *SLIT3* (Zhou et al., 2011) (Fig. 4D). To verify the accuracy of the RNA-seq results, expression levels of these four hub mRNAs were analyzed *via* qRT-PCR in the eight EP and eight NP samples. *SLIT3* expression was significantly lower in the EP group than in the NP group (Fig. 5A). However, there were no significant differences in expression levels of the remaining three hub mRNAs between the two groups (Fig. 5A). IHC was performed to explore the cellular localization of *SLIT3* in villous tissue and to further verify differences in expression between EP and NP (Fig. 5B). IHC scores for *SLIT3* were significantly lower in patients with EP than in the NP controls (Fig. 5B), consistent with the RNA-seq results. Taken together, the results of the RNA-seq bioinformatic analysis, qRT-PCR validation, and IHC confirmed that *SLIT3* may be involved in regulating angiogenesis of EP villi. Next, we validated the target gene *ROBO4* (Xiao et al., 2022), which is downstream of* SLIT3* and reportedly associated with angiogenesis, *via* qRT-PCR. This assay indicated that relative expression of *ROBO4* was statistically lower in EP than in NP villous tissue (*p* = 0.007) (Fig. S4).

### Validation of *SLIT3*-mediated angiogenesis promotion

To further investigate whether *SLIT3* promoted angiogenesis, we performed sprouting assays to visualize capillary development using HUVECs (Zahra et al., 2019). Briefly, we carried out an angiogenesis experiment by culturing HUVECs into spheroids (sprouting assay) then adding *SLIT3* to the basal medium. Vascular sprout formation was strongly induced by addition of the positive control VEGF (Fig. 5C), indicating that this platform was powerful for examining angiogenesis. *SLIT3* addition significantly induced vascular sprouting compared to unconditioned medium (Fig. 5C), indicating that *SLIT3* promoted angiogenesis and that low *SLIT3* expression resulted in poor vascular development within the EP placental villi.

### Validation of hub miRNAs targeting *SLIT3*

**Figure 6:**
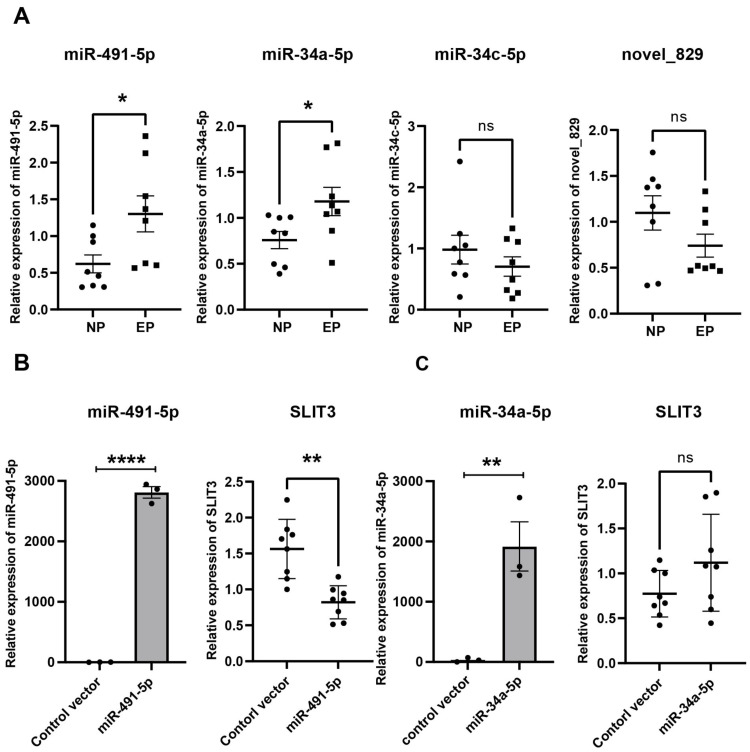
Validation of hub miRNA targeting *SLIT3*. (A) Four candidate miRNAs found to potentially target *SLIT3* were validated in villi from the IP and EP groups *via* qRT-PCR (*n* = 8 per group). (B) HTR-8 cell lines transfected with a miR-491-5p overexpression plasmid showed a marked increase in miR-491-5p expression 72 h after transfection compared with the control vector group. Overexpression of miR-491-5p resulted in a significant decrease in expression of *SLIT3*. HTR-8 cell lines transfected with a miR-34a-5p overexpression plasmid showed a significant increase in miR-34a-5p expression 72 h after transfection compared with the control vector group, but there was no significant change in *SLIT3* expression. ^∗^*p* < 0.05, ^∗∗^*p* < 0.01, ^∗∗∗∗^*p* < 0.0001, ns, no significance. Data are represented as mean ± SEM.

From the miRNA-mRNA regulatory network, four candidate miRNAs were found to potentially target *SLIT3*, namely miR-491-5p, miR-34a-5p, miR-34c-5p, and novel_829 (Fig. 4C). qRT-PCR showed that miRNA-491-5p and miRNA-34a-5p expression levels were significantly higher in EP villi than in the NP group. No significant differences in expression levels of miRNA-34c-5p or novel-829 were observed between the two groups (Fig. 6A). To verify whether these miRNAs directly inhibited *SLIT3*, miR-491-5p and miR-34a-5p were each overexpressed in the HTR-8 cell line, then *SLIT3* expression was measured. As expected, miR-491-5p and miR-34a-5p were significantly up-regulated in cells transfected with the miR-491-5p and miR-34a-5p overexpression vectors, respectively (Fig. 6B). Overexpression of miR-491-5p resulted in a significant decrease in *SLIT3* expression (Fig. 6B), whereas miR-34a-5p overexpression was not associated with a significant change in *SLIT3* expression (Fig. 6B). Furthermore, a dual-luciferase reporter assay showed that miR-491-5p could bind the 3′ UTR of *SLIT3* mRNA (Fig. S3A). Simultaneously, a significant increase in *SLIT3* expression after transfection with the miR-491-5p mimic was revealed in an RNA-pull down assay (Fig. S3B), further suggesting that miR-491-5p could directly target and regulate *SLIT3*.

### Correlations between *SLIT3* expression and *β*-HCG levels, *β*-HCG levels and vascular number, and *SLIT3* expression and vascular number

Analysis of *SLIT3* expression (as quantified by RNA-seq) and *β*-HCG levels revealed a positive correlation between the two parameters (*p* = 0.03, *r* = 0.68) (Fig. S2B). This was subsequently confirmed in a larger sample size (*n* = 18, *p* = 0.02, *r* = 0.53) (Fig. S2C). Interestingly, further analysis of the same samples also verified positive correlations between *β*-HCG levels and the number of villous capillaries (*p* < 0.001, *r* = 0.80) (Fig. S2D) and between *SLIT3* expression and vascular number (*p* = 0.02, *r* = 0.56) (Fig. S2E).

## Discussion

In this study, we found that the mean and total cross-sectional areas of placental villi were significantly increased in EP patients compared to NP patients. The number of capillaries per unit area was significantly reduced in EP villi compared with NP controls, and was positively correlated with *β*-HCG levels. miRNA-491-5p-*SLIT3* was also identified as involved in regulation of villous angiogenesis. Moreover, a positive correlation was confirmed between relative *SLIT3* expression and vascular number.

Placental villous capillaries play a vital role in the transfer of nutrients, oxygen, and metabolites between maternal and fetal blood (Jirkovska et al., 2012). Compared to decidual angiogenesis, less is known about villous angiogenesis, which is a critical factor in functional placental formation and embryonic growth. It has previously been reported that the branching patterns of villous capillaries and structural alterations in placental villi are associated with gestational diabetes mellitus, preeclampsia, and FGR (Egbor et al., 2006; Jirkovska et al., 2012). Goddijn et al. (2000) indicated that there was no association between EP villous capillaries and chromosomal abnormalities. However, no previous studies have compared differences in villus morphology and capillary number between EP and NP samples.

Our results demonstrated that, compared to NP villi, EP villi tended to have a larger mean cross-sectional area, but fewer capillaries per unit area, and lower *β*-HCG levels. This indicated that EP is associated with a low potential to enlarge the villous surface and the vascular network that are key in maternofetal transport and embryonic growth. Unlike the endometrium, the epithelium of the fallopian tubes is poorly decidualized, which affects production and secretion of VEGF and vascular establishment of the embryo (Nowacek et al., 1999; Torry et al., 1996; Torry & Torry, 1997). The increase in villus cross-sectional area may be a compensation for the poor environment in which the embryo grows.

*SLIT*s comprise a highly conserved family of secreted proteins that were originally discovered in the nervous system (Zhou et al., 2013a). Three *SLIT* genes (*SLIT1, SLIT2,* and *SLIT3*) have been identified in mammals. Slit signaling plays a vital role in axon guidance (Yu et al., 2014), angiogenesis (Zhou et al., 2011), and cell migration (Qi et al., 2014) *via* the *Robo* receptor. *SLIT/ROBO* systems have also been found in a variety of non-neuronal tissues, including the lung (Anselmo et al., 2003), kidney (Piper et al., 2000), ovary (Dickinson, Myers & Duncan, 2008), and placenta (Li et al., 2015; Liao et al., 2010). In those with preeclampsia, the *SLIT/ROBO* system is abnormally expressed in placental vascular endothelial cells and trophoblast cells, affecting placental development and function by altering the function of trophoblast cells and endothelial cells (Liao et al., 2012). These findings suggest that *SLIT/ROBO* signaling plays a vital role in placental angiogenesis and function during NP. Another study revealed that *SLIT2* is expressed in extravillous trophoblasts (EVTs) of tubal pregnancy, and that changes in *SLIT2* expression are linked to vascular remodeling of the fallopian tube (Li et al., 2015). *SLIT3* is a new angiogenic factor that binds to *ROBO4*, promoting endothelial cell proliferation and migration and inducing angiogenesis *in vivo* (Xiao et al., 2022; Zhang et al., 2009). Moreover, expression of *ROBO4* in placental arteries and veins (Huminiecki et al., 2002) and of *SLIT3* in the placenta (Dickinson et al., 2004) have been previously reported. Our results revealed that relative expression levels of both *SLIT3* and *ROBO4* were decreased in EP villous tissue compared to NP, and that *SLIT3* did promote angiogenesis, consistent with the poor angiogenesis of EP villi. EP is known to have faulty decidualization compared to NP, and it is uncertain whether altered *SLIT3* expression in EP villi is associated with poor tubal decidualization.

Interestingly, *SLIT3* expression was positively correlated with the number of villous capillaries and levels of *β*-HCG, and *β*-HCG levels were positively correlated with the number of villous capillaries. This confirmed our hypothesis that *SLIT3* may be associated with the poor villous angiogenesis of tubal EP. It is unclear how *SLIT3* regulates *β*-HCG levels, due to a lack of evidence in the literature, but our results led us to a hypothesis. In the human placenta, there are three major trophoblast subpopulations: CTBs, EVTs, and syncytiotrophoblast (STBs); of these three types, *β*-HCG is secreted only by STBs (Handschuh et al., 2009). Because early gestational trophoblasts are supplied with nutrients by the intravillous capillaries, we hypothesize that *SLIT3* promotes trophoblast growth by enhancing villous capillary growth, which in turn increases *β*-HCG secretion. However, this hypothesis requires further verification. In addition, higher *β*-HCG levels (a result of high trophoblast activity) can increase the risk of tubal pregnancy rupture and decrease the likelihood of conservative treatment (Fukami et al., 2016). *SLIT3* expression in the villous tissue is positively correlated with *β*-HCG levels, implying that high *SLIT3* expression in EP villous tissue may be associated with poor prognosis of tubal pregnancy, possibly through promotion of angiogenesis in the villi. Thus, *SLIT3* is a promising prognostic factor for tubal pregnancy and a potential therapeutic target for reducing the risk of EP.

In cancer cells, miR-491-5p induces apoptosis and suppresses proliferation and invasion (Denoyelle et al., 2014; Zhou et al., 2013b); in lung cancer, it restrains angiogenesis (Yang et al., 2021). miR-491-5p is also reportedly expressed in placental tissue and has potential roles in trophoblast proliferation, invasion, and angiogenesis (Liu et al., 2020a). Compared with normal placental tissue, miR-491-5p is upregulated in placental tissue collected from patients with preeclampsia, and overexpression suppresses trophoblast invasion and vascular remodeling (Liu et al., 2020a). Similarly, in the present study, miR-491-5p was expressed at higher levels in villous tissues from women with tubal EP compared to those with NP. Furthermore, miR-491-5p was found to suppress expression of *SLIT3*. We therefore speculate that miR-491-5p affected villous angiogenesis by targeting *SLIT3* in villi, which may be associated with the mechanism underlying villous dysplasia in the fallopian tube environment. This hypothesis requires confirmation in further functional experiments.

There are several limitations of this study that should be noted. First, the sample size was relatively small. The correlation between *SLIT3* and villous capillaries will be further confirmed by expanding the sample size in the future, with the aim of providing new biomarkers for predicting abnormalities in villi and villous vascular development. Additionally, although HTR8/SVneo is widely used as a model to study the invasion, migration, metabolism and other functions of placental trophoblast cells (Basak et al., 2018; Tan et al., 2022; Yin et al., 2022), it cannot fully reflect the real situation of trophoblast cells *in vivo* due to its own limitations; therefore, primary trophoblast cells should be used to further validated the findings of this study in the future. Furthermore, the specific molecular mechanisms by which miR-491-5p and *SLIT3* regulate EP have not been fully investigated. Validation and functional experiments with larger sample sizes are urgently needed to elucidate the pathological mechanisms of villous dysplasia in EP.

## Conclusion

Our results revealed that the morphology, capillary density, and transcriptome expression profiles of EP villi differed from those of NP villi. These changes may have been related to the biological roles of miR-491-5p and *SLIT3*. In addition, *SLIT3* was identified as a putative predictor of abnormal chorionic villus development. However, its true functional significance requires further verification.

##  Supplemental Information

10.7717/peerj.14850/supp-1 Supplemental Information 1 Supplementary methods

10.7717/peerj.14850/supp-2 Supplemental Information 2 Kyoto Encyclopedia of Genes and Genomes (KEGG) pathways of the 103 overlapping genes

10.7717/peerj.14850/supp-3 Supplemental Information 3 The rate of proliferative CTB in villi for EP and NP and correlation analysis (A) The rate of proliferative CTB in villi was quantified for EP (*n* = 8) and NP (*n* = 8). Similar proliferative activity of stem cell populations was observed between the two types of placental villi. Scale bar = 100 µm. Data are represented as mean ± SEM, ns: no significance. (B) Spearman’s rank correlation test revealed that the relative expression of *SLIT3* (as quantified by RNA-seq) was positively correlated with *β*-HCG levels. (C) The positive correlation between SLIT3 and *β*-HCG was confirmed in a subsequent validation with a larger sample size (n NP = 10, n EP = 8). (D) The same samples also confirmed a positive correlation between *β*-HCG levels and the number of villous capillaries. (E) *SLIT3* expression was positively correlated with the number of villous capillaries.

10.7717/peerj.14850/supp-4 Supplemental Information 4 MiR-491-5p directly regulates the expression of SLIT3 (A). Luciferase expression levels of HEK293 cells transfected with control or WT SLIT3 3′UTR or mutant SLIT3 3′UTR vector plus miR-491-5p mimics or control . MiR-491-5p inversely modulated the luciferase activity of plasmids containing WT 3′-UTR of SLIT3′showing that miR-491-5p could combine with 3′UTR of SLIT3 mRNA. (B). RNA-pull down assay. SLIT3 mRNA expression increased significantly following transfection with miR-491-5p mimics. This further illustrates that miR-491-5p can targetedly regulate SLIT3. ^∗^*p* < 0.05, ^∗∗^*p* < 0.01, ^∗∗∗∗^*p* < 0.0001, ns, no significance.

10.7717/peerj.14850/supp-5 Supplemental Information 5 Validation of *SLIT3* downstream genes in NP and EP villi Validation of the SLIT3 receptor (ROBO4) in the villi of EP ( *n* = 8) and NP (*n* = 5) groups via qRT-PCR. Data are represented as mean ± SEM. ^∗^*p* < 0.05, ns, no significance.

10.7717/peerj.14850/supp-6 Supplemental Information 6 Demographic characteristics of NP and EP

10.7717/peerj.14850/supp-7 Supplemental Information 7 Primers for miRNAs

10.7717/peerj.14850/supp-8 Supplemental Information 8 Primers for mRNAs

10.7717/peerj.14850/supp-9 Supplemental Information 9 Detective results of CNVs and contamination in ten samples

10.7717/peerj.14850/supp-10 Supplemental Information 10 The information of 49 DE-miRNAs between the NP and EP

10.7717/peerj.14850/supp-11 Supplemental Information 11 The DE-mRNAs between the NP and EP

10.7717/peerj.14850/supp-12 Supplemental Information 12 Raw data for Figs. 2, 5 and 6

## Additional Information and Declarations

### Data Availability

The following information was supplied regarding data availability: The data is available at GEO: GSE210031, GSE183319.
