# Supplementary material for: Abnormal expression of SLIT3 induces intravillous vascularization dysplasia in ectopic pregnancy
Source: PeerJ. 2023 Feb 10;11:e14850. doi: 10.7717/peerj.14850 (PMC9924138; doi:10.7717/peerj.14850)
Supplement: Supplemental Information 1 [file peerj-11-14850-s001.docx]

**Abnormal expression of *SLIT3* induces intravillous vascularization dysplasia in ectopic pregnancy**

Qian Zhu^1,2, §^, Xiaoya Zhao^1,2, §^, Duo Zhang^1,2^, Wei Xia^1,2^, Jian Zhang^1,2, *^

1 Department of Obstetrics and Gynecology, International Peace Maternity and Child Health Hospital, School of Medicine, Shanghai Jiaotong University,

No. 910, Hengshan Rd, Shanghai 200030, China

2 Shanghai Municipal Key Clinical Specialty, Shanghai, China

§ Co-first author

Qian Zhu and Xiaoya Zhao contributed equally to this study.

* Corresponding author:

Jian Zhang, Department of Obstetrics and Gynecology, International Peace Maternity and Child Health Hospital, School of Medicine, Shanghai Jiao Tong University,

Shanghai, China. Email: [zhangjian_ipmch@sjtu.edu.cn](mailto:zhangjian_ipmch@sjtu.edu.cn)

***Supplementary method***

**RNA extraction and quality control**

15 villus samples (7 EP and 8 NP) were collected for miRNA-seq and mRNA-seq analysis. Total RNA was extracted from the villus samples using TRIzol (Invitrogen, Waltham, MA, USA) according to the manufacturer’s recommendations. RNA purity was checked using the kaiaoK5500® Spectrophotometer (Kaiao, Beijing, China). RNA integrity and concentration were assessed using the RNA Nano 6000 Assay Kit and the Agilent Bioanalyzer 2100 system (Agilent Technologies, Santa Clara, CA, USA).

**Study design**

To exclude chromosomal abnormalities and maternal cell contamination, the 15 villus samples (7 EP and 8 NP) were examined for copy number variation (CNV), RNA quality, and maternal cell contamination. Ten samples (5 EP and 5 NP) were finally selected for miRNA-seq and mRNA-seq analyses. In addition, 16 villus samples (8 EP and 8 NP) were selected to verify the sequencing results.

**MiRNA sequencing**

All samples passed quality control thresholds and were run on an array together within the same batch. Approximately 3 μg of RNA from each sample was used for cDNA library construction following the protocol provided with the NEBNext® Multiplex Small RNA Library Prep Set for Illumina (NEB, Ipswich, MA, USA). We utilized the special structure of small RNA (i.e., the complete phosphate group at the 5' end and hydroxyl group at the 3' end) to directly add the linker to the ends, followed by reverse transcription to cDNA. Next, the cDNA library was obtained after polymerase chain reaction (PCR) amplification and PAGE. Raw data (raw reads) in fastq format were processed using custom Perl and Python scripts. In this step, clean data (clean reads) were obtained by removing reads containing poly-N sequences and 5′ adapters, reads lacking a 3′ adapter or the insert tag, reads containing poly-A or T or G or C, and low-quality reads. The Q30 values and GC-content were calculated.

The small RNA tags were mapped to the reference sequence using Bowtie [[1](#_ENREF_1)] without mismatches to analyze their expression and physical distribution. Mapped small RNA tags were used to search against miRBase20.0 for known miRNA prediction. Modified mirdeep2 [[2](#_ENREF_2)] and srna-tools-cli were used to obtain the potential miRNAs and to draw secondary structures. The characteristics of the hairpin structure of the miRNA precursor can be used to predict novel miRNAs. miREvo [[3](#_ENREF_3)] and mirdeep2 [[4](#_ENREF_4)] were integrated to predict novel miRNAs based on the secondary structure, the Dicer cleavage site, and the minimum free energy of the small RNA tags unannotated in the former steps.

**Identification and target gene prediction of DE-miRNAs**

MiRNA expression levels were estimated by TPM (transcript per million million) as follows [[5](#_ENREF_5)]: Normalized expression = mapped read count/Total reads × 1000000. Levels in the two groups were compared using the DESeq R Bioconductor package (1.8.3) [<http://www.r-project.org/>]. The P-values were adjusted using the Benjamini & Hochberg method. False discovery rate (FDR) < 0.05 and |log2(FoldChange)| ≥ 0.6 were set as the thresholds for DE-miRNA identification by default. DE-miRNAs were visualized in a volcano plot and heatmap generated using the gplots [[6](#_ENREF_6)] and ggplot2 [[7](#_ENREF_7)] R Bioconductor packages.

Target genes of DE-miRNAs were predicted using miRanda [[8](#_ENREF_8)] and RNAhybrid (Miranda_Score ≥ 150, Miranda_Energy < -20, RNAhybrid_Energy < -25). Only targets identified by both programs were selected for further analyses.

**mRNA sequencing and identification of DE-mRNAs**

Sequencing libraries were generated using the NEBNext® Ultra™ RNA Library Prep Kit for Illumina (#E7530L; NEB) following the manufacturer’s recommendations and index codes were added to attribute sequences to samples.

The library fragments were purified using QiaQuick PCR Kits (Qiagen, Hilden, Germany) followed by elution with EB buffer, terminal repair, A-tailing and adapter addition. The target products were retrieved, PCR was performed, and the library was completed. After cluster generation, the libraries were sequenced on an Illumina platform to generate 150 bp paired-end reads. The original data (raw data) were filtered to guarantee the data quality as follows: trim Smart-seq2 public primer sequence from the reads, remove reads containing more than 5 bp of adapter sequences, discard reads containing >5% unknown nucleotides (N) and low-quality reads. The reference genomes and annotation files were downloaded from ENSEMBL (http://www.ensembl.org/index.html). Bowtie2 v2.2.3 was used to build the genome index, and clean data were then aligned to the reference genome using HISAT2 v2.1.0. Gene expression levels were calculated by fragments per kilobase per million mapped reads (FPKM), defined as follows: FPKM = cDNA fragments/Mapped Reads (Million) × Transcript Length (kb)).

A count matrix was examined using the DESeq2 [[9](#_ENREF_9), [10](#_ENREF_10)] R Bioconductor package to normalize count data, estimate dispersion, and fit a negative binomial model for each gene. Compared with the control, genes with |log2(FoldChange)| ≥ 1 and FDR < 0.05 were identified as differentially expressed genes (DE-mRNAs). The DE-mRNAs were visualized in a volcano plot and heatmap generated using the gplots and ggplot2 R Bioconductor packages.

**Intersection of target mRNAs and DE-mRNAs**

To narrow the scope of key genes, the predicted target genes of DE-miRNAs obtained using miRanda were compared with DE-mRNAs. The Venny website (<https://bioinfogp.cnb.csic.es/tools/venny/index.html>) was used to obtain the intersection between the DE-miRNA target genes and DE mRNAs. Based on the hypothesis that miRNAs negatively regulate target mRNAs, only the interactions of DE-miRNAs and DE-mRNAs with opposite expression changes were included in subsequent analyses.

**Functional annotation and pathway enrichment analysis**

The intersecting genes then were subjected to functional enrichment analyses using Gene Ontology (GO) and Kyoto Encyclopedia of Genes and Genomes (KEGG) implemented in the clusterProfiler R Bioconductor package. A value of FDR < 0.05 was considered statistically significant.

**Construction of a miRNA–mRNA network**

A miRNA–mRNA regulatory network was constructed using Cytoscape 3.4.0.

**Retrovirus production**

The precursor sequences of hsa-miR-491-5p (premiR-491-5p) and hsa-miR-34a-5p (premiR-34a-5p) were obtained from the miRBase database. After replacing U with T, the sequence was as follows, respectively: TTGACTTAGCTGGGTAGTGGGGAACCCTTCCATGAGGAGTAGAACACTCCTTATGCAAGATTCCCTTCUACCTGGCTGGGTTGG, GGCCAGCTGTGAGTGTTTCTTTGGCAGTGTCTTAGCTGGTTGTTGTGAGCAATAGTAAGGAAGCAATCAGCAAGTATACTGCCCTAGAAGTGCTGCACGTTGTGGGGCCC. Double restriction digestion of the vector, ligation of the expression vector and the target fragment, transformation and sequencing identification were all completed by Sangon Biotech Co., Ltd. (Shanghai, China). The validated vector and the retrovirus packaging vectors (G·P and VSVG) were cotransfected into 293T cells using Lipofectamine 2000 reagent. After 48 and 72 hours, virus particles were harvested and then purified.

**Reference**

[1] B. Langmead, C. Trapnell, M. Pop, S.L. Salzberg, Ultrafast and memory-efficient alignment of short DNA sequences to the human genome, Genome Biology 10(3) (2009) R25.

[2] M.R. Friedlander, S.D. Mackowiak, N. Li, W. Chen, N. Rajewsky, miRDeep2 accurately identifies known and hundreds of novel microRNA genes in seven animal clades, Nucleic Acids Res 40(1) (2012) 37-52.

[3] M. Wen, Y. Shen, S. Shi, T. Tang, miREvo: an integrative microRNA evolutionary analysis platform for next-generation sequencing experiments, BMC Bioinformatics 13 (2012) 140.

[4] H.J. Wu, Y.K. Ma, T. Chen, M. Wang, X.J. Wang, PsRobot: a web-based plant small RNA meta-analysis toolbox, Nucleic Acids Res 40(Web Server issue) (2012) W22-8.

[5] L. Zhou, J. Chen, Z. Li, X. Li, X. Hu, Y. Huang, X. Zhao, C. Liang, Y. Wang, L. Sun, M. Shi, X. Xu, F. Shen, M. Chen, Z. Han, Z. Peng, Q. Zhai, J. Chen, Z. Zhang, R. Yang, J. Ye, Z. Guan, H. Yang, Y. Gui, J. Wang, Z. Cai, X. Zhang, Integrated profiling of microRNAs and mRNAs: microRNAs located on Xq27.3 associate with clear cell renal cell carcinoma, PLoS One 5(12) (2010) e15224.

[6] G. Warnes, B. Bolker, L. Bonebakker, R. Gentleman, W. Huber, A. Liaw, T. Lumley, M. Mächler, A. Magnusson, S. Möller, gplots: Various R programming tools for plotting data, 2005.

[7] H. Wickham, ggplot2: elegant graphics for data analysis, Springer2016.

[8] A.J. Enright, B. John, U. Gaul, T. Tuschl, C. Sander, D.S. Marks, MicroRNA targets in Drosophila, Genome Biol 5(1) (2003) R1.

[9] S. Anders, W. Huber, Differential expression analysis for sequence count data, Genome Biol 11(10) (2010) R106.

[10] M.I. Love, W. Huber, S. Anders, Moderated estimation of fold change and dispersion for RNA-seq data with DESeq2, Genome Biol 15(12) (2014) 550.
