# Supplementary figures and images for: Abnormal expression of SLIT3 induces intravillous vascularization dysplasia in ectopic pregnancy
Source: PeerJ. 2023 Feb 10;11:e14850. doi: 10.7717/peerj.14850 (PMC9924138; doi:10.7717/peerj.14850)

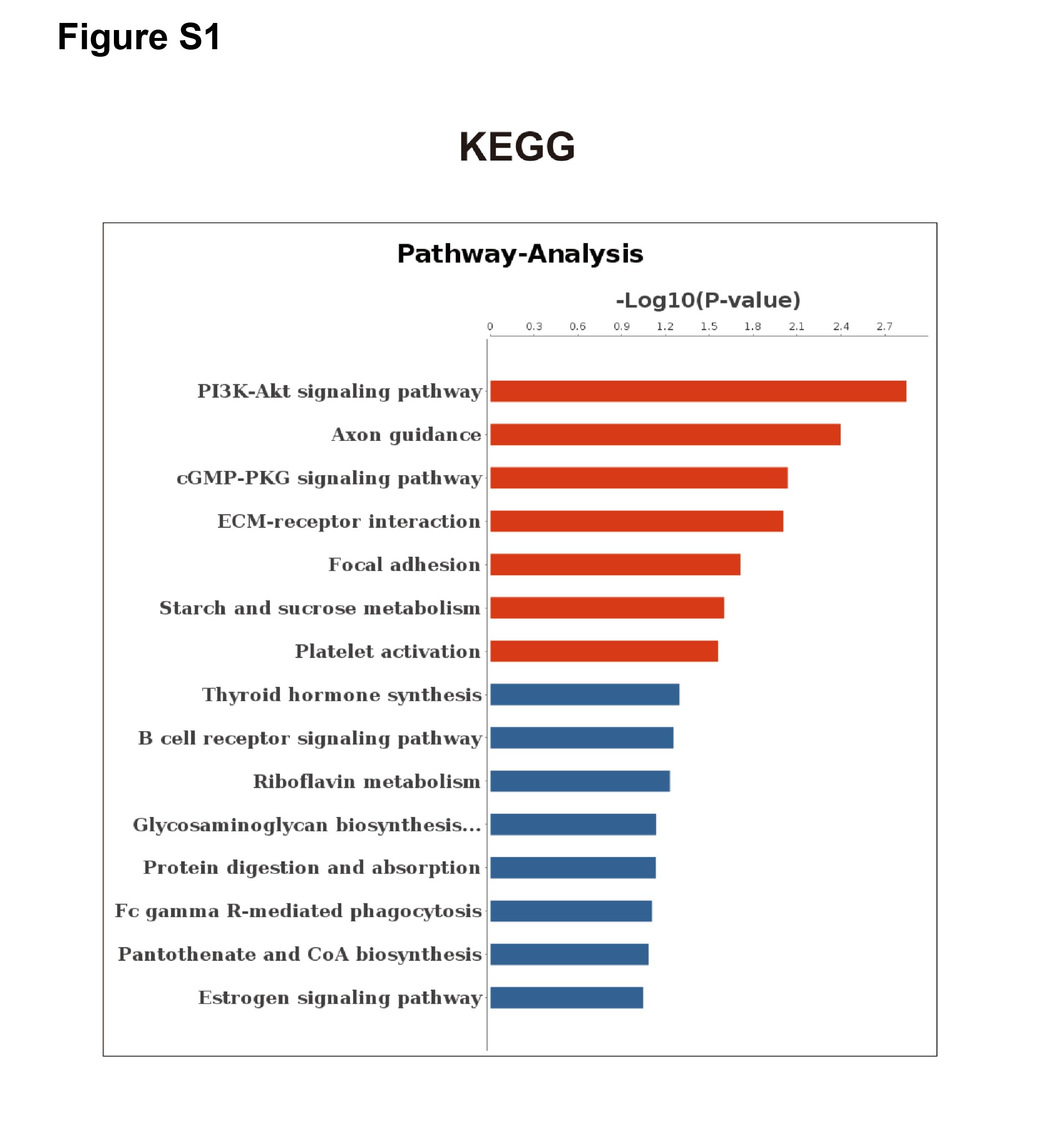

Supplement: Supplemental Information 2 [file peerj-11-14850-s002.jpg]

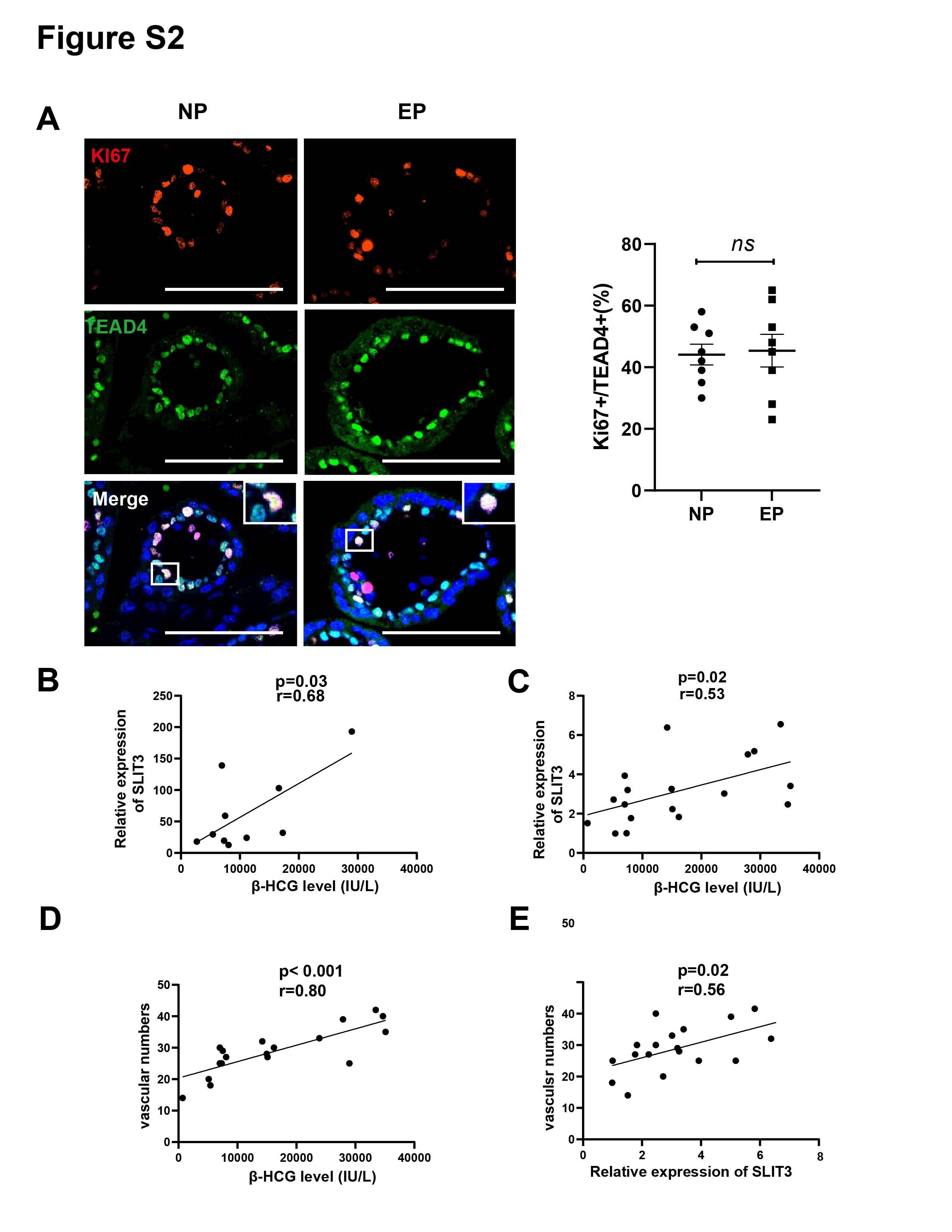

Supplement: Supplemental Information 3 — (A) The rate of proliferative CTB in villi was quantified for EP (n = 8) and NP (n = 8). Similar proliferative activity of stem cell populations was observed between the two types of placental villi. Scale bar = 100 µm. Data are represented as mean ± SEM, ns: no significance. (B) Spearman’s rank correlation test revealed that the relative expression of SLIT3 (as quantified by RNA-seq) was positively correlated with β-HCG levels. (C) The positive correlation between SLIT3 and β-HCG was confirmed in a subsequent validation with a larger sample size (n NP = 10, n EP = 8). (D) The same samples also confirmed a positive correlation between β-HCG levels and the number of villous capillaries. (E) SLIT3 expression was positively correlated with the number of villous capillaries. [file peerj-11-14850-s003.jpg]

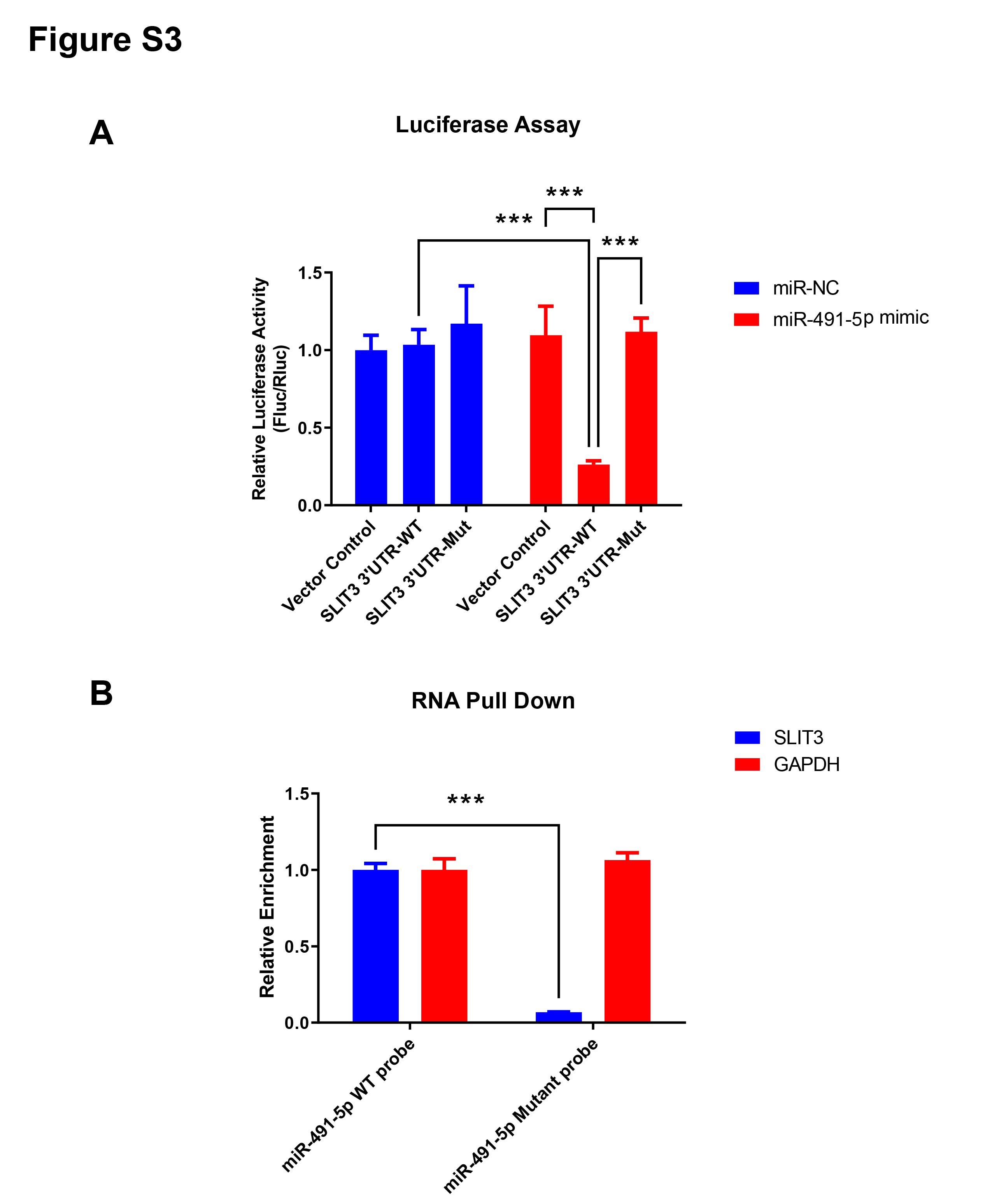

Supplement: Supplemental Information 4 — (A). Luciferase expression levels of HEK293 cells transfected with control or WT SLIT3 3′UTR or mutant SLIT3 3′UTR vector plus miR-491-5p mimics or control . MiR-491-5p inversely modulated the luciferase activity of plasmids containing WT 3′-UTR of SLIT3′showing that miR-491-5p could combine with 3′UTR of SLIT3 mRNA. (B). RNA-pull down assay. SLIT3 mRNA expression increased significantly following transfection with miR-491-5p mimics. This further illustrates that miR-491-5p can targetedly regulate SLIT3. ∗p < 0.05, ∗∗p < 0.01, ∗∗∗∗p < 0.0001, ns, no significance. [file peerj-11-14850-s004.jpg]

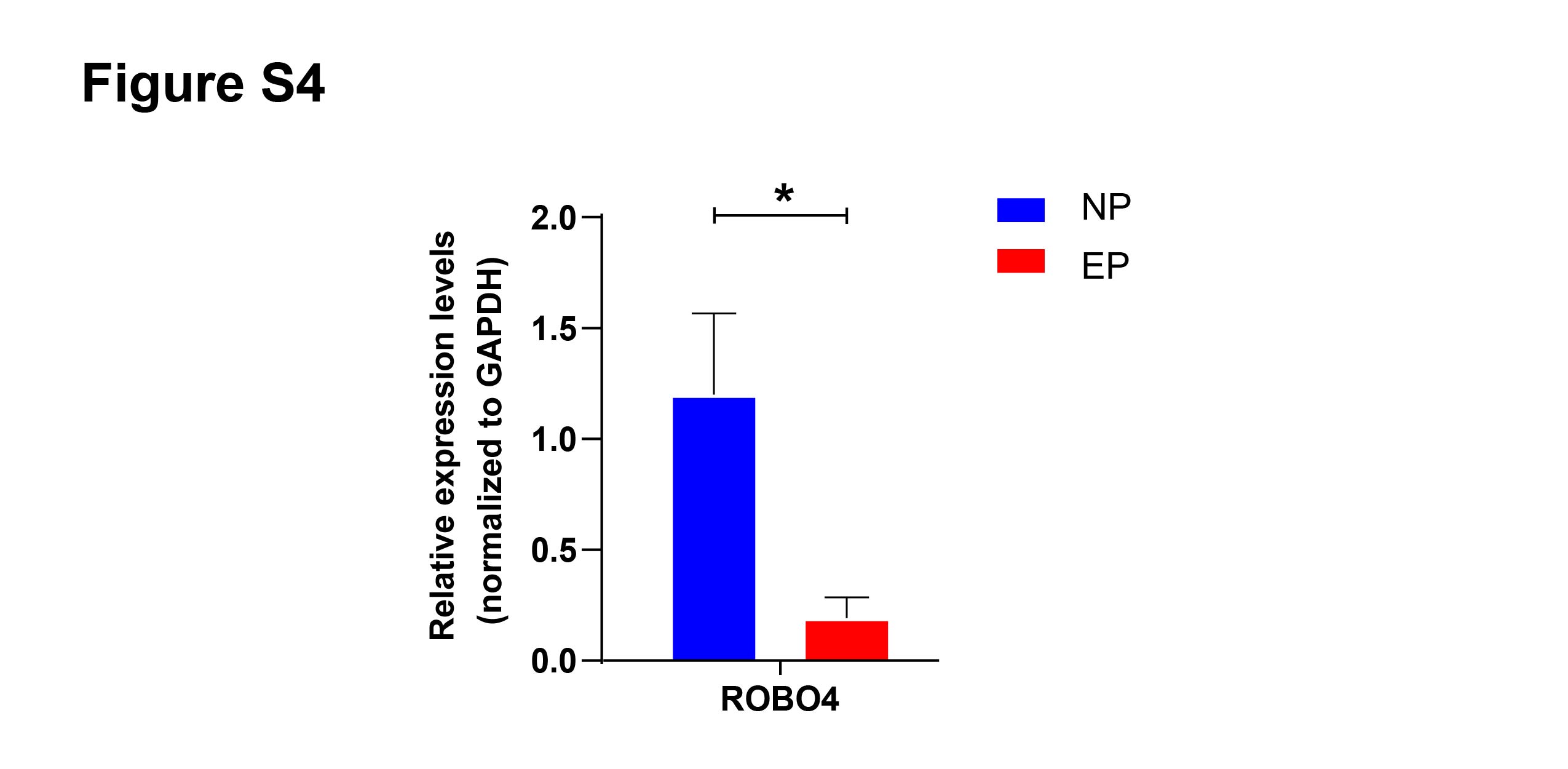

Supplement: Supplemental Information 5 — Validation of the SLIT3 receptor (ROBO4) in the villi of EP ( n = 8) and NP (n = 5) groups via qRT-PCR. Data are represented as mean ± SEM. ∗p < 0.05, ns, no significance. [file peerj-11-14850-s005.jpg]
